# Efficacy and Safety of Sodium Hyaluronate Injection for Moderate‐To‐Severe Neck Wrinkles: A Multicenter, Single‐Blind, Randomized, Parallel‐Controlled Trial

**DOI:** 10.1111/jocd.71135

**Published:** 2026-08-27

**Authors:** Ji‐Na Zheng, Jianming Yang, Kai Yang, Zhiyu Liu, Qian Yu, Xiaoke Liu, Xiuli Li, Shuqin Zhang, Man Hu, Hongyue Diao, Julien Cle'ment, Shiliang Wang, Yuxin Hou, Hengtao Li, Yuna Luo, Kun Chen, Jiajia Liu, Yougai Huang, Weiwei Li, Yan Liu, Yuling Shi, Jun Gu, Yu Gong

**Affiliations:** ^1^ Department of Dermatology Shanghai Tenth People's Hospital, Tongji University School of Medicine Shanghai China; ^2^ Institute of Psoriasis Tongji University School of Medicine Shanghai China; ^3^ Department of Plastic Surgery Beijing Tsinghua Changgung Hospital Beijing China; ^4^ Department of Medical Aesthetics Beijing Jishuitan Hospital, Capital Medical University Beijing China; ^5^ Laboratories Hyamed SA Plan‐les‐Ouates Switzerland; ^6^ Hyamed Biotechnology (Zhuhai) Co., Ltd Zhuhai People's Republic of China; ^7^ Department of Dermatology Shanghai Skin Disease Hospital, Tongji University School of Medicine Shanghai China

**Keywords:** efficacy, moderate‐to‐severe neck wrinkles, multicenter, RCT, safety, sodium hyaluronate injection

## Abstract

**Background:**

Hyaluronic acid (HA) occupies the micro‐environment of the dermal matrix, filling depressions and imparting a smooth appearance to the skin. HA fillers are widely used for wrinkle correction. Still, the efficacy and safety of ANN0032, a new HA‐based injection, have not yet been assessed in clinical trials for moderate to severe neck wrinkles.

**Objective:**

To evaluate the efficacy and safety of ANN0032, a novel HA‐based injection, for the correction of moderate‐to‐severe horizontal neck wrinkles.

**Methods:**

In this multicenter, randomized, single‐blind, parallel‐controlled trial, 156 subjects were randomized (1:1) to receive either ANN0032 treatment (*n* = 78) or an established HA filler Hearty (*n* = 78). The primary efficacy endpoint was the proportion of subjects achieving a ≥ 1‐point improvement on the Allergan Transverse Neck Lines Scale (ATNLS) at Week 4 posttreatment, as assessed by blinded evaluators.

**Results:**

At Week 4, ANN0032 demonstrated non‐inferior improvement compared with Hearty, with 83.3% versus 71.8% of subjects achieving the primary endpoint (rate difference, 11.54%; 95% CI: −3.29% to 26.37%). Both groups showed comparable safety profiles with no serious adverse events reported.

**Conclusion:**

ANN0032, a HA‐based filler, demonstrated non‐inferior efficacy and a favorable safety profile compared to an established HA filler, Hearty, in improving moderate‐to‐severe neck wrinkles.

## 1 Introduction

Horizontal neck lines, a prominent manifestation of cutaneous aging, have emerged as a key aesthetic concern due to their profound impact on facial harmony and psychosocial well‐being. The neck's anatomical vulnerability is characterized by thin dermis, diminished collagen density, and high exposure to extrinsic aging factors such as ultraviolet radiation and repetitive mechanical [1, 2]. These lines are exacerbated by repetitive downward gaze associated with the frequent use of digital devices. The demand for cosmetic procedures beyond the face has steadily increased, with patients frequently requesting aesthetic improvements of the neck to achieve a harmonious appearance. Moderate‐to‐severe neck wrinkles often lead to dissatisfaction with self‐image, contributing to psychological distress and a growing demand for minimally invasive cosmetic interventions [3, 4].

Although various treatment modalities—such as ultrasound, radiofrequency (monopolar or bipolar), microneedling, intense pulsed light, and ablative lasers—have been employed for neck rejuvenation, most offer only modest improvement in static transverse neck lines and typically require multiple sessions [5]. Botulinum toxin type A is effective for dynamic platysmal bands but shows limited efficacy for static horizontal lines and is associated with low patient satisfaction [6, 7, 8]. Surgical approaches improve skin laxity and contour but have little effect on deep etched wrinkles. Deep ablative laser resurfacing, while effective on the face, poses safety concerns for the neck due to thinner skin and reduced adnexal structures which delay healing and increase the risk of scarring [9].

Until recently, assessment of neck wrinkles lacked standardized grading tools. The introduction of the validated 5‐point Transverse Neck Line Scale (TNLS) has enabled more consistent clinical evaluation, with a 1‐point reduction representing a clinically meaningful improvement.

Hyaluronic acid (HA)–based fillers have demonstrated substantial success in facial rejuvenation due to their biocompatibility, hydration capacity, and ability to restore volume and stimulate collagen synthesis [10, 11, 12, 13]. Sodium hyaluronate (SH), a non‐cross‐linked form of HA, is widely used for facial wrinkles but lacks robust evidence for its efficacy on neck lines [14]. However, evidence supporting its use for neck wrinkles remains scarce, with most studies focusing on facial applications or combining SH with other modalities [15]. Existing clinical trials on neck wrinkle treatments are limited by small sample sizes, single‐center designs, or lack of controlled comparisons [16]. Furthermore, the biomechanical properties of neck skin differ markedly from facial skin, necessitating tailored approaches to ensure optimal efficacy and safety [17]. A rigorous evaluation of SH injections in this anatomically distinct region is critical to address gaps in clinical practice guidelines.

This multicenter, single‐blind, randomized, parallel‐controlled trial aims to evaluate the efficacy and safety of ANN0032, a novel sodium hyaluronate injection, in treating moderate‐to‐severe horizontal neck wrinkles. Hearty, a non‐cross‐linked HA compound with proven effects for rejuvenation of neck wrinkles, served as the active control. The primary efficacy endpoint was the proportion of subjects achieving a ≥ 1‐point improvement on the Allergan Transverse Neck Lines Scale (ATNLS) at Week 4 posttreatment. By applying a standardized outcome measure and a rigorous controlled design, this study seeks to provide robust evidence to guide clinical practice and expand understanding of HA‐based therapies in the understudied cervical region.

## 2 Materials and Methods

### 2.1 Subjects

Eligible participants were male or female aged 18–65 years with moderate‐to‐severe transverse neck wrinkles, defined by a score of 2 or 3 on ATNLS between February 17, 2023, and June16, 2023. All subjects were required to comply with study procedures as outlined in the protocol.

Exclusion criteria included: pregnancy or lactation, planned pregnancy during the study period; significant scarring or active skin disease (e.g., infection, psoriasis, herpes simplex) in the neck region; cancerous or precancerous lesions, or unhealed wounds on the neck; previous neck treatments (e.g., botulinum toxin, laser resurfacing, chemical peels, dermabrasion) within 24 weeks before enrollment; use of immunosuppressive agents; anticoagulant or antiplatelet therapy within 2 weeks before injection; coagulation disorders; prior head and neck radiotherapy within the past year; history of keloid or hypertrophic scarring; severe dysfunction in major organ systems (e.g., cardiovascular, hepatic, renal, pulmonary, neurologic); autoimmune or immunodeficiency disorders; participation in other clinical trials within 4 weeks before screening; history of severe allergic reactions, known hypersensitivity to HA products, or multiple drug allergies. Subjects deemed at high risk or unsuitable due to unrealistic expectations, potential confounding factors, or psychological disorders were excluded.

### 2.2 Study Design and Ethical Aspects

This was a multicenter, randomized, single‐blind (independent evaluator‐blinded), parallel‐controlled trial conducted at three clinical sites in China. Participants were randomized in a 1:1 ratio to receive either ANN0032 or Hearty. Randomization was stratified by site using a centralized system. Efficacy assessments were conducted by independent evaluators who were blinded to treatment allocation, whereas subjects and injecting investigators were not blinded due to practical constraints related to treatment administration. This study was approved by the institutional ethics committees of three independent participating centers. The trial was registered in the regional medical device clinical trial registry. The study was conducted in accordance with the Declaration of Helsinki. Written informed consent was obtained from all participants before study enrollment.

### 2.3 Treatments

ANN0032 is a sterile injectable solution containing 32 mg/mL of sodium hyaluronate in phosphate‐buffered saline (PBS), supplied in a 2 mL single‐use syringe. The PBS vehicle contains sodium dihydrogen phosphate monohydrate, disodium hydrogen phosphate dihydrate, sodium chloride, and water for injection. The control group received Hearty (IMEIK, China), an established HA‐based injectable product approved by the National Medical Products Administration of China for neck wrinkle rejuvenation.

### 2.4 Injection Procedure

All injections were performed by trained investigators experienced in aesthetic injection techniques. The study products were administered using a linear threading technique into the dermal layer along the horizontal neck lines. The needle was inserted at an angle of approximately 15 degrees with the bevel facing upward, and the product was delivered intradermally while withdrawing the needle along the direction of the neck lines.

For each injection pass, 0.1–0.2 mL of product was administered, and injections were continued until adequate filling of the target neck line was achieved. Each subject received two treatment sessions, with an interval of 4 weeks between sessions.

The injection volume was adjusted according to the severity and extent of neck wrinkles. The total injection volume per individual neck line did not exceed 2.0 mL per session, and the total injection volume per treatment session did not exceed 8.0 mL. Injection procedures were standardized across study centers to ensure consistency and reproducibility.

### 2.5 Trial Procedure

The study included screening (V0), treatment visits (V1–V3), and follow‐up assessments (V4–V7). At V0, informed consent, demographic data, medical history, vital signs, pregnancy testing, and laboratory tests were obtained. ATNLS scoring and photographic documentation were performed at screening, pre‐ and posttreatment, and at all follow‐up visits. Randomization occurred at V1, followed by product administration at V1 and V3. Subject diaries were used to record posttreatment symptoms and compliance. Follow‐ups were scheduled at Weeks 2, 4, 12, and 24 posttreatments (with allowable visit windows of ±2 to ±15 days) (Table 1).

**TABLE 1:** Trial procedure.

Trial procedure	Screening period	The first treatment period	The follow‐up period of the first treatment	The follow‐up period of the first treatment/The final treatment period	The follow‐up period of the last treatment			
Follow‐up	V0	V1	V2	V3	V4	V5	V6	V7
Time	−14 to 0 days	0 day	2 weeks ± 2 days	4 weeks ± 5 days	After the last treatment			
					2 weeks ± 5 days	4 weeks ± 5 days	12 weeks ± 7 days	24 weeks ± 15 days
Sign informed consent	▲							
Demographic data	▲							
History collection	▲							
Basic vital signs and body weight	▲	▲	▲	▲	▲	▲	▲	▲
Laboratory tests	▲				▲			▲
Neck photo collection		▲ Before randomization and immediately after treatment		▲		▲	▲	▲
Pregnancy test	▲							▲
Verification of screening criteria	▲	▲						
Random		▲						
ANTLS scoring	▲							
Blind independent ANTLS scoring		▲ Before randomization		▲		▲	▲	▲
Injection therapy		▲		▲				
Subject Diary Card		▲	▲	▲	▲			
Researcher GAIS evaluation				▲		▲	▲	▲
Subjects GAIS evaluation				▲		▲	▲	▲
Devices defect		▲		▲				
Expected AE		▲	▲	▲	▲	▲	▲	▲
Records of AE and SAE	▲	▲	▲	▲	▲	▲	▲	▲
Combined medication/devices and concomitant treatment	▲	▲	▲	▲	▲	▲	▲	▲

Abbreviations: AE, adverse event; ANTLS, Allergan Transverse Neck Lines Scale; GAIS, Global Aesthetic Improvement Scale; SAE, Serious Adverse Event.

### 2.6 Evaluation

Neck wrinkle severity was assessed using the Allergan Transverse Neck Lines Scale (ATNLS), a validated 5‐point photonumeric scale specifically developed for the evaluation of transverse neck wrinkles (0 = none, 1 = mild, 2 = moderate, 3 = severe, 4 = extreme). The scale has been widely used in clinical studies of neck rejuvenation and demonstrates good inter‐rater reliability and clinical validity. A ≥ 1‐point improvement on the ATNLS has been established as a clinically meaningful change and was therefore selected as the responder definition for the primary efficacy endpoint in this study. The primary endpoint was to assess the improvement rate of neck wrinkles assessed by blinded evaluators using ATNLS at Week 4 post‐final injection by independent blinded evaluators. Two independent evaluators performed assessments independently. When discrepancies occurred between their evaluations, a third independent blinded evaluator reviewed the case and provided the final adjudicated score to ensure objectivity and consistency of the efficacy assessment.

Secondary Endpoints were to assess the improvement rate in neck wrinkle severity, assessed by blinded evaluators. Improvement score in neck wrinkle severity—assessed by blinded evaluators. Global Aesthetic Improvement Scale (GAIS) evaluation—assessed separately by subjects and injecting investigators, who were not blinded to treatment allocation [18].

### 2.7 Statistical Analysis

All quantitative data were expressed as mean ± standard deviation (SD), and categorical data were summarized as frequencies or percentages. Data normality was assessed prior to applying parametric tests. Statistical analysis was performed using the SPSS software (Version 23.0; IBM Corp., Chicago, IL, USA). Between‐group comparisons of continuous variables were performed using independent‐sample *t*‐tests. Chi‐squared or Fisher's exact tests were used for categorical variables. For non‐inferiority testing, conclusions were based on comparison of the two‐sided 95% confidence interval with the prespecified non‐inferiority margin. For other exploratory comparisons, a *p*‐value < 0.05 was considered statistically significant.

The non‐inferiority margin was prespecified as an absolute difference of −10% in the responder rate (proportion of subjects achieving ≥ 1‐point improvement on the ATNLS at Week 4 after the final treatment). This margin was selected a priori based on clinical judgment and interpretability for responder‐based aesthetic endpoints: a difference within 10 percentage points is generally considered unlikely to represent a clinically meaningful loss of benefit for patients when both treatments yield high responder rates and when a ≥ 1‐point improvement on a validated severity scale is regarded as clinically meaningful. Accordingly, ANN0032 would be concluded non‐inferior to the active control if the lower bound of the two‐sided 95% confidence interval (ANN0032 minus control) was greater than −10%, consistent with recommended reporting principles for non‐inferiority randomized trials.

## 3 Results

### 3.1 Randomization and Baseline Characteristics

A total of 156 subjects were screened, among whom 153 met the inclusion criteria and were randomized in a 1:1 ratio to receive either ANN0032 (*n* = 78) or Hearty (*n* = 78). Of these, 151 subjects completed the study. Three participants voluntarily withdrew, and two were discontinued due to poor compliance and inadequate follow‐up. Demographic and baseline characteristics were well balanced between groups, with no statistically significant differences observed (Table 2).

**TABLE 2:** Demographics and baseline characteristics.

	Total (*N* = 156)	ANN0032 (*N* = 78)	Hearty (*N* = 78)	*p*
Age, years				
N (Missing)	156 (0)	78 (0)	78 (0)	0.90^b^
Mean (SD)	36.51 (7.40)	36.42 (6.26)	36.60 (8.43)	
Median	36.00	36.50	35.50	
Q1, Q3	31.00, 41.00	31.00, 41.00	31.00, 41.00	
Min. Max	20.00, 61.00	25.00, 59.00	20.00, 61.00	
Gender				
Male, *n* (%)	11 (7.1)	8 (10.3)	3 (3.8)	0.21^a^
Female, *n* (%)	145 (92.9)	70 (89.7)	75 (96.2)	
Nationality				
Han, *n* (%)	145 (92.9)	74 (94.9)	71 (91.0)	0.53^a^
Other, *n* (%)	11 (7.1)	4 (5.1)	7 (9.0)	
Height, cm				
N (Missing)	156 (0)	78 (0)	78 (0)	0.21^b^
Mean (SD)	163.56 (6.26)	163.99 (5.84)	163.12 (6.65)	
Median	163.00	163.50	162.00	
Q1, Q3	159.00, 167.00	160.00, 167.00	158.00, 166.00	
Min, Max	152.00, 187.00	153.00, 181.00	152.001, 87.00	
Weight, kg				
N (Missing)	156 (0)	78 (0)	78 (0)	0.45^b^
Mean (SD)	60.02 (9.71)	60.47 (9.59)	59.57 (9.87)	
Median	58.25	59.25	58.00	
Q1, Q3	53.00, 65.00	53.80, 66.00	52.00, 65.00	
Min, Max	42.10, 96.00	44.00, 86.00	42.10, 96.00	
BMI (Kg/m^2^)				
N (Missing)	156 (0)	78 (0)	78 (0)	0.60^b^
Mean (SD)	22.38 (3.01)	22.41 (2.86)	22.35 (3.17)	
Median	21.76	21.76	21.67	
Q1, Q3	20.25, 23.94	20.72, 24.01	19.88, 23.88	
Min, Max	16.86, 31.89	17.19, 31.89	16.86, 30.30	

^a^ *t*‐test.

^b^ Wilcoxon rank sum test, C‐chi‐square test, D‐Fisher's accuracy method.

### 3.2 Primary Efficacy Endpoint

**FIGURE 1:**
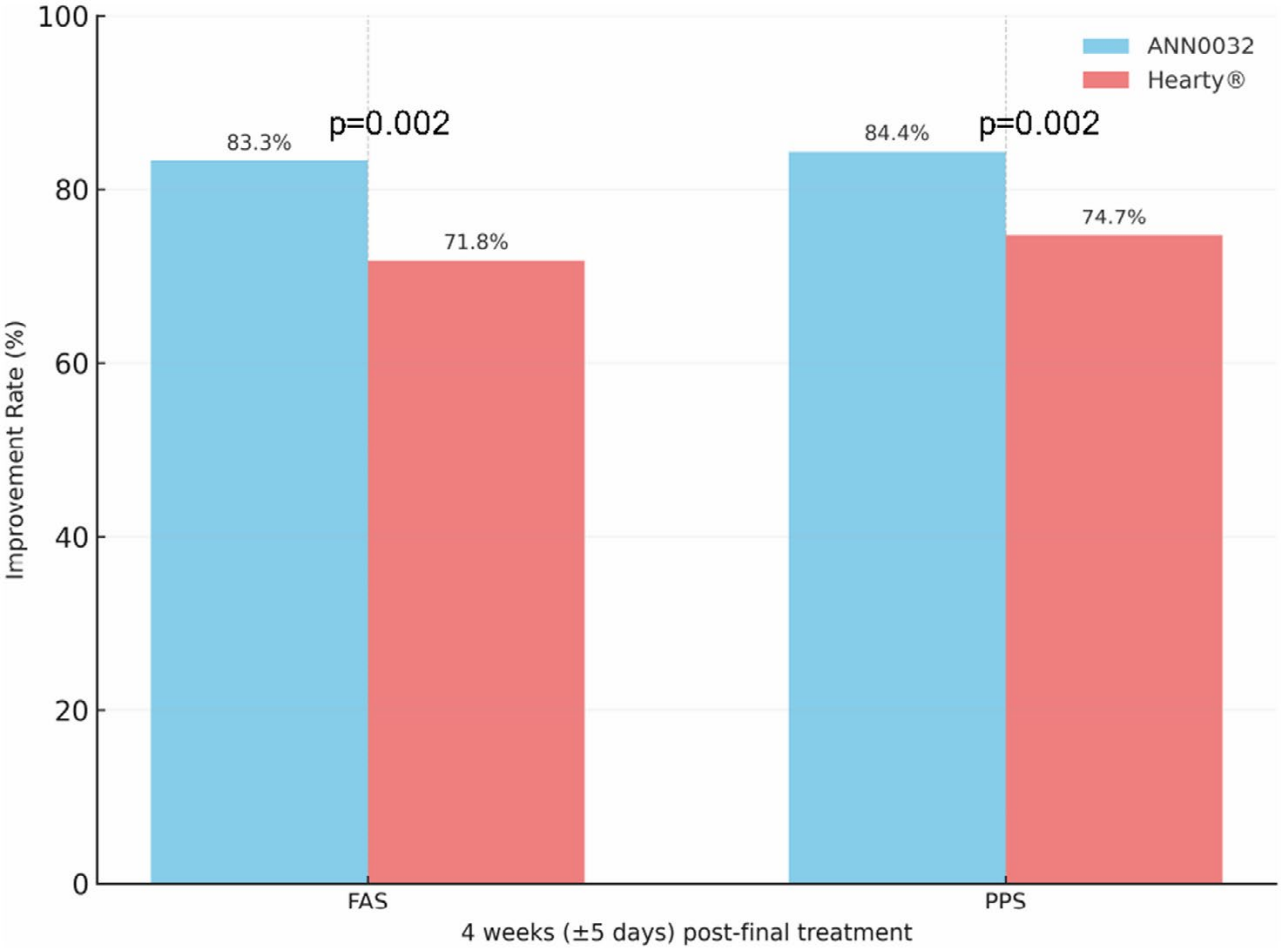
Primary efficacy endpoint: Neck wrinkle improvement rate at 4 weeks post‐final treatment. Bar chart comparing the proportion of subjects achieving ≥ 1‐point reduction on the ATNLS in the ANN0032 and Hearty groups, based on both FAS and PPS populations.

At 4 weeks (±5 days) after the final treatment, the full analysis set (FAS) analysis showed the proportion of subjects achieving a ≥ 1‐point reduction in ATNLS score was 83.3% (65/78) in the ANN0032 group and 71.8% (56/78) in the Hearty group. Because the lower bound of the two‐sided 95% confidence interval for the between‐group difference was greater than the prespecified non‐inferiority margin of −10%, non‐inferiority of ANN0032 to the active control was established (Figure 1). The Per Protocol Set (PPS) analysis yielded consistent findings: the improvement rate was 84.4% (65/77) in the experimental group and 74.7% (56/75) in the control group, with a rate difference of 9.75% (95% CI: −4.83% to 24.33%) (Figure 1). Similarly, the lower limit of the confidence interval surpassed the non‐inferiority margin of −10%, thereby establishing non‐inferiority of the experimental group (Table S1). These results confirm that the improvement rate of the experimental group is non‐inferior to that of the control group under both FAS and PPS analyses. These results demonstrate that the improvement rate of the experimental group is statistically non‐inferior to that of the control group.

### 3.3 Secondary Efficacy Endpoint

**FIGURE 2:**
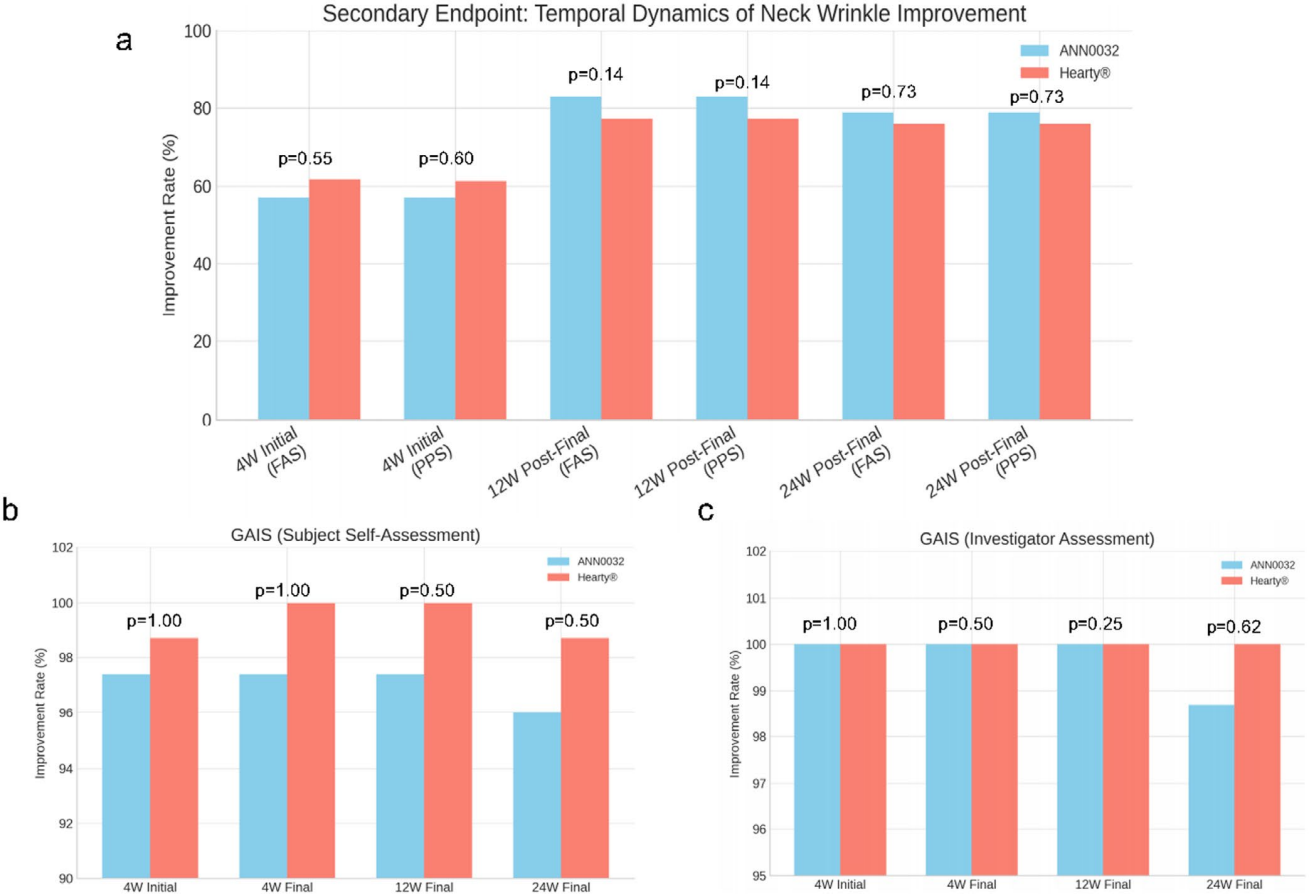
(a) Temporal dynamics of neck wrinkle improvement rate across follow‐up time points. Improvement rates were evaluated at 4 weeks after initial injection, and at 4, 12, and 24 weeks after the final injection. No statistically significant differences were observed between the groups. (b) The Global Aesthetic Improvement Scale (GAIS) was assessed by subjects. Subject self‐assessment showed high improvement rates (> 96%) in both treatment groups across all timepoints. (c) The Global Aesthetic Improvement Scale (GAIS) was assessed by investigators. Investigator ratings demonstrated nearly universal improvement in both groups, with > 98% improvement at all timepoints.

Figure 2 presents the analysis results for the secondary efficacy endpoint, defined as the improvement rate of neck wrinkles. Both FAS and PPS analyses demonstrated no statistically significant differences between the experimental and control groups at the following time points: Week 4 (±5 days) following the initial treatment, Week 4 (±5 days) post‐final treatment, Week 12 (±7 days) post‐final treatment, and Week 24 (±15 days) post‐final treatment (Table S2). The analysis results for the secondary efficacy endpoint, neck wrinkle improvement score, are presented in Table S3. Both FAS and PPS analyses demonstrated no statistically significant differences between the experimental and control groups (Table S3). Figure 2b presents the analysis results for the secondary efficacy endpoint, the improvement rate of the GAIS based on subject self‐assessment. Both FAS and PPS analyses demonstrated no statistically significant differences in GAIS improvement rates between the experimental and control groups at the following time points: Week 4 (±5 days) following the initial treatment, Week 4 (±5 days) post‐final treatment, Week 12 (±7 days) post‐final treatment, and Week 24 (±15 days) post‐final treatment (Table S4). Figure 2c presents the analysis results of the improvement rate of the GAIS, based on investigator assessment. Both FAS and PPS analyses demonstrated no statistically significant differences between the experimental and control groups at the following time points: Week 4 (±5 days) following the initial treatment, Week 4 (±5 days) post‐final treatment, Week 12 (±7 days) post‐final treatment, and Week 24 (±15 days) post‐final treatment (Table S5).

### 3.4 Adverse Events

The safety outcomes are summarized in Table 3, with adverse events presented as both the total number of events and the number of subjects experiencing at least one event. In the ANN0032 group, 33 subjects (42.3%) experienced at least one adverse event, compared with 31 subjects (39.7%) in the Hearty group, with no statistically significant difference between groups. Serious adverse events were infrequent and comparable between groups, and no device‐related adverse events, discontinuations due to adverse events, or fatal adverse events were reported.

**TABLE 3:** Summary of adverse events (total number of events and number of subjects affected).

	ANN0032 (*N* = 78)	Hearty (*N* = 78)	*p*
All AEs			
Number of events	231	250	
Subjects with ≥ 1 AE, *n* (%)	33 (42.3%)	31 (39.7%)	0.74
SAEs			
Number of events	1	3	
Subjects with ≥ 1 AE, *n* (%)	1 (1.3%)	3 (3.8%)	0.62
Discontinuations due to AEs, *n* (%)	0 (0.0%)	0 (0.0%)	
Fatal AEs, *n* (%)	0 (0.0%)	0 (0.0%)	
Device‐Related AEs, *n* (%)	0 (0.0%)	0 (0.0%)	

*Note:* Adverse events (AEs) are summarized as both the total number of events and the number of subjects experiencing at least one event. Between‐group comparisons were performed based on the number of subjects with ≥ 1 event using the chi‐squared test or Fisher's exact test, as appropriate. Device‐related adverse events were defined as those assessed as possibly or definitely related to the study device.

## 4 Discussion

**FIGURE 3:**
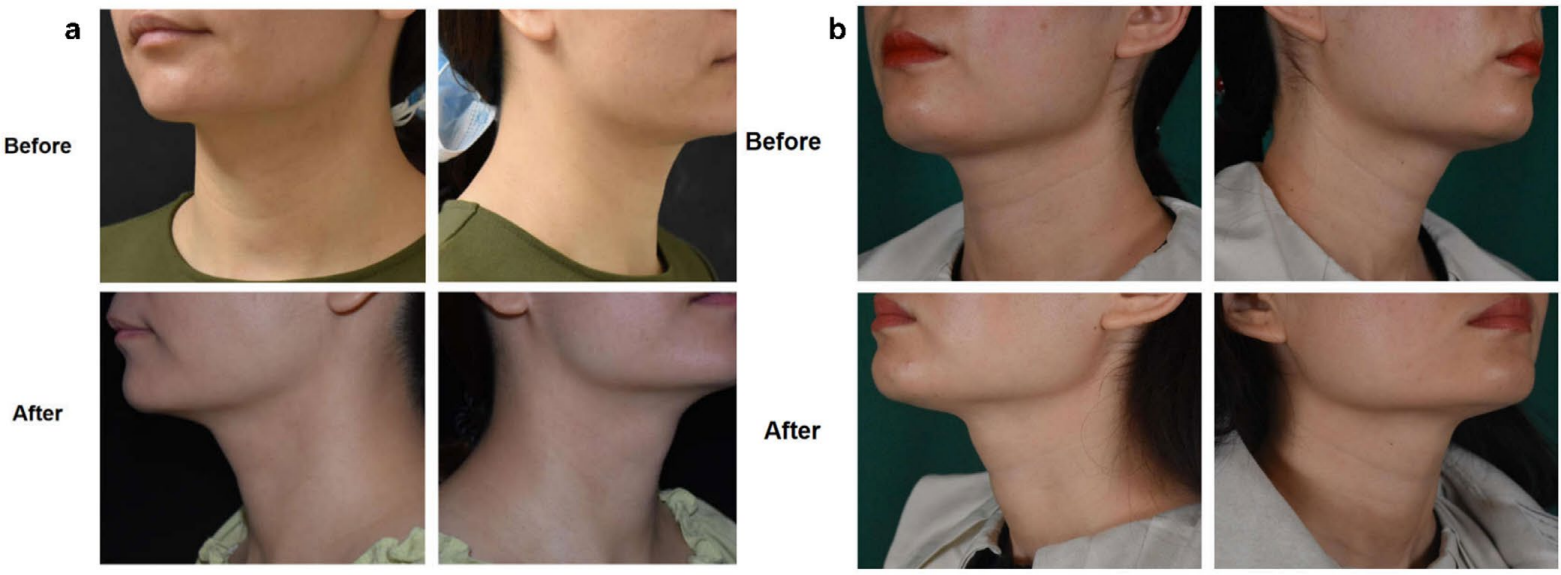
(a) Representative clinical outcomes of ANN0032 injection (Case 1). Standardized photographs of a female subject before (top row) and 4 weeks after (bottom row) completing ANN0032 treatment. Images demonstrate a visible reduction in horizontal neck lines and improved skin texture. (b) Representative clinical outcomes of ANN0032 injection (Case 2). Standardized before‐and‐after images showing significant attenuation of moderate‐to‐severe neck wrinkles following ANN0032 treatment. The subject's skin appears smoother with enhanced dermal elasticity.

This multicenter, randomized controlled trial demonstrated the non‐inferiority of sodium hyaluronate ANN0032 compared to the established HA filler Hearty in the treatment of moderate‐to‐severe neck wrinkles, with comparable safety profiles. The primary efficacy endpoint, assessed via blinded evaluation using the ATNLS at Week 4 posttreatment, revealed non‐inferior improvement for ANN0032 compared with Hearty, with an improvement rate of 83.3% versus 71.8% (rate difference, 11.54%; 95% CI: −3.29% to 26.37%). These findings were robust across both FAS and PPS analyses, reinforcing the reliability of the results despite minor participant attrition. The consistency between FAS and PPS outcomes underscores the minimal impact of protocol deviations on the study's conclusions, aligning with recommendations for rigorous non‐inferiority trial designs [19]. Figure 3 illustrates representative clinical outcomes from subjects treated with ANN0032, demonstrating a visible reduction in neck wrinkles from baseline to posttreatment.

Although subject‐reported GAIS improvement rates were numerically higher in the control group at several follow‐up time points, no statistically significant differences were observed between groups in either the FAS or PPS analyses. This divergence between subject‐reported and evaluator‐based outcomes is not unexpected in aesthetic clinical trials and may reflect the inherently subjective nature of patient‐reported measures. Similar discrepancies between patient‐reported and evaluator‐assessed outcomes have been described in previous aesthetic studies, underscoring the importance of interpreting patient‐reported measures as complementary rather than definitive indicators of comparative efficacy. Although GAIS assessments were not blinded, the primary efficacy endpoint was evaluated exclusively by independent blinded evaluators using a validated scale, thereby minimizing the risk of assessment bias for the primary outcome. These results suggest that ANN0032 achieves comparable aesthetic outcomes to Hearty in both short‐ and medium‐term follow‐ups. The alignment of subject‐reported GAIS with investigator assessments further supports the clinical relevance of treatment effects, as patient perception remains a key determinant of success in aesthetic procedures [18]. Figure 3 further exemplifies the aesthetic outcomes observed in a separate cohort of subjects, reinforcing the visual consistency and patient satisfaction following ANN0032 treatment.

The safety profile of ANN0032 was favorable with AE incidence rates (42.3% vs. 39.7%) and SAEs (1.3% vs. 3.8%) comparable to the control group. Notably, no device‐related AEs, discontinuations, or fatal events occurred, consistent with the established safety of HA‐based fillers in facial applications [10]. The absence of severe device‐related complications aligns with prior studies emphasizing the biocompatibility and low immunogenicity of stabilized HA formulations [14]. These findings support the use of ANN0032 as a safe alternative for neck rejuvenation, extending the evidence base for HA fillers beyond traditional facial indications. Notably, the neck's vascular anatomy—proximity to the carotid artery and jugular vein—has raised concerns about embolism risk. However, no vascular events occurred in this trial, likely due to standardized injection techniques (e.g., linear threading in the superficial dermis) and clinician expertise, underscoring the importance of anatomical training for injectors.

The anatomical uniqueness of neck skin—thinner dermis, reduced collagen, and dynamic mechanical stress—has historically limited the extrapolation of facial filler data to this region [20]. Our study addresses this gap by directly evaluating HA efficacy in neck‐specific contexts, a critical advancement given the increasing demand for minimally invasive neck treatments. The non‐inferior performance of ANN0032 may reflect its optimized rheological properties (e.g., elasticity, cohesivity) tailored to withstand neck movement, as hypothesized by Sundaram et al. in their analysis of HA biophysics [17].

While this trial achieved its primary objective, several limitations warrant consideration. First, the 24‐week follow‐up period may be insufficient to capture long‐term efficacy and safety, particularly given the dynamic nature of neck aging. Extended studies are needed to evaluate durability beyond 6 months. Second, the single‐blind design, though pragmatic, introduces potential bias in patient‐reported outcomes. Future trials could employ double‐blinding to further reduce subjectivity. Third, the study population lacked demographic diversity; broader inclusion across skin types and age groups is necessary to enhance generalizability. Lastly, given the non‐inferiority design, superiority cannot be claimed; head‐to‐head trials are warranted to explore potential advantages of ANN0032 in specific subpopulations or treatment contexts.

## 5 Conclusion

This trial establishes sodium hyaluronate ANN0032 as a non‐inferior, safe, and clinically effective option for treating moderate‐to‐severe neck wrinkles. Its efficacy and safety profile, combined with favorable patient satisfaction, make it a valuable addition to the therapeutic arsenal for neck rejuvenation. Future studies should investigate longer‐term outcomes, explore underlying mechanisms such as HA‐induced collagen remodeling, and compare ANN0032 within multimodal therapeutic regimens to further define its role in aesthetic dermatology.

## Author Contributions

Study conception and design: Yu Gong, Jun Gu, Yuling Shi. Data acquisition: Ji‐Na Zheng, Jianming Yang, Kai Yang, Zhiyu Liu, Qian Yu, Xiaoke Liu, Xiuli Li, Shuqin Zhang, Man Hu, Hongyue Diao, Yuxin Hou, Hengtao Li, Yuna Luo, Kun Chen, Jiajia Liu, Yougai Huang, Weiwei Li, Yan Liu. Data analysis and interpretation: Ji‐Na Zheng, Julien Clément, Shiliang Wang. Drafting of manuscript: Ji‐Na Zheng. Critical revision of manuscript: Yu Gong, Jun Gu, Yuling Shi. All authors reviewed and approved the final manuscript and agree to be accountable for all aspects of the work.

## Funding

This work was supported by grants from the National Key Research and Development Program of China (No. 2023YFC2508106); the National Natural Science Foundation of China (Nos. 82173405, 82473517, 82430101, and 82273510); the Innovation Program of Shanghai Municipal Education Commission (No. 2025GDZKZD06); the Shanghai Dermatology Research Center (No. 2023ZZ02017); and the Clinical Research Plan of SHDC (No. SHDC22022302).

This study was supported by the grants awarded to Yu Gong from Shanghai Health Science Talent Ability Improvement Project (JKKPYC‐2023‐B08). This study was supported by the Shanghai Shenkang Hospital Development Center Project for the Promotion and Optimization of Diagnosis and Treatment Technologies (No. SHDC22025306), Clinical Research Plan of SHDC.

## Disclosure

The authors declare that no generative artificial intelligence tools were used in the preparation, writing, data analysis, image generation, image processing, interpretation of results, or revision of this manuscript. All contents of the manuscript were prepared, reviewed, and approved by the authors.

## Ethics Statement

The authors confirm that this study was conducted in accordance with the Declaration of Helsinki and Good Clinical Practice guidelines. Ethical approval was obtained from the Institutional Review Boards of all participating centers. Written informed consent was obtained from all participants prior to enrollment. This study was approved by the institutional ethics committees of three independent participating centers (Approval No. SHSY‐IEC‐5.0/22G33/P01; No. 22555‐2‐02; No. Jilun [L2022] No. 022‐00). The trial was registered in the regional medical device clinical trial registry (Registration No. Yuexie Linbei 20220544).

## Consent

Written informed consent was obtained from all participants before enrollment. In addition, written informed consent was obtained from all patients for the publication of clinical photographs.

## Conflicts of Interest

The authors declare no conflicts of interest.

## Supporting information


**Table S1:** Primary efficacy endpoint: The improvement rate of neck wrinkles at 4 weeks (±5 days) post‐final treatment.

**Table S2:** Secondary efficacy endpoint: Temporal dynamics of neck wrinkle improvement rate.

**Table S3:** Secondary efficacy endpoint: Temporal dynamics of neck wrinkle improvement score.

**Table S4:** Secondary efficacy endpoint: Temporal dynamics of the GAIS improvement rate based on subject self‐assessment.

**Table S5:** Secondary efficacy endpoint: Temporal dynamics of the GAIS improvement rate based on investigator self‐assessment.

## Data Availability

The data that support the findings of this study are available from the corresponding author upon reasonable request.
