# Supplementary material for: Efficacy and Safety of Sodium Hyaluronate Injection for Moderate‐To‐Severe Neck Wrinkles: A Multicenter, Single‐Blind, Randomized, Parallel‐Controlled Trial
Source: J Cosmet Dermatol. 2026 Aug 27;25(9):e71135. doi: 10.1111/jocd.71135 (PMC13522398; doi:10.1111/jocd.71135)
Supplement: Supplementary file 2 [file JOCD-25-e71135-s002.docx]

**Supplementary table 2. Secondary efficacy endpoint-Temporal dynamics of neck wrinkle improvement rate**

|  |  | **FAS** |  | **PPS** |  |
| --- | --- | --- | --- | --- | --- |
| **Indicator** | **Efficacy** | **ANN0032 (N = 78)** | **Hearty®**  **(N = 78)** | **ANN0032 (N = 78)** | **Hearty®**  **(N = 78)** |
| **Week 4 (±5 days) following the initial treatment** | **Improved, n(%)** | 44(57.1) | 47(61.8) | 44(57.1) | 46(61.3) |
|  | **Unimproved, n (%)** | 33(42.9) | 29(38.2) | 33(42.9) | 29(38.7) |
|  | **Intergroup difference in improvement rate and 95%CI** | -4.70% (-20.24%-10.84%) | | -4.19% (-19.8%-11.42%) | |
|  | **Test statistic** | 0.35 | | 0.28 | |
|  | **P-value** | 0.55 | | 0.60 | |
| **Week 4 (±5 days) post-final treatment** | **Improved, n(%)** | 65(84.4) | 56(74.7) | 65(84.4) | 56(74.7) |
|  | **Unimproved, n (%)** | 12(15.6) | 19(25.3) | 12(15.6) | 19(25.3) |
|  | **Intergroup difference in improvement rate and 95%CI** | 9.75%(-3.00%-22.50%) | | 9.75% (3.00%-22.50%) | |
|  | **Test statistic** | 2.22 | | 2.22 | |
|  | **P-value** | 0.14 | | 0.14 | |
| **Week 12 (±7 days) post-final treatment** | **Improved, n(%)** | 63(82.9) | 58(77.3) | 63(82.9) | 58(77.3) |
|  | **Unimproved, n (%)** | 13(17.1) | 17(22.7) | 13(17.1) | 17(22.7) |
|  | **Intergroup difference in improvement rate and 95%CI** | 5.56%(-7.14%-18.27%) | | 5.56% (-7.14%-18.27%) | |
|  | **Test statistic** | 0.73 | | 0.73 | |
|  | **P-value** | 0.39 | | 0.39 | |
| **Week 24 (±15 days) post-final treatment** | **Improved, n(%)** | 60(78.9) | 57(76.0) | 60(78.9) | 57(76.0) |
|  | **Unimproved, n (%)** | 16(21.1) | 18(24.0) | 16(21.1) | 18 (24.0) |
|  | **Intergroup difference in improvement rate and 95%CI** | 2.95%(-10.37%-16.27%) | |  | |
|  | **Test statistic** | 0.19 | | 0.19 | |
|  | **P-value** | 0.66 | | 0.66 | |

Note: Response rates between the two groups were compared using the chi-square test or Fisher’s exact test; missing data were not imputed.
