# Supplementary material for: Efficacy and Safety of Sodium Hyaluronate Injection for Moderate‐To‐Severe Neck Wrinkles: A Multicenter, Single‐Blind, Randomized, Parallel‐Controlled Trial
Source: J Cosmet Dermatol. 2026 Aug 27;25(9):e71135. doi: 10.1111/jocd.71135 (PMC13522398; doi:10.1111/jocd.71135)
Supplement: Supplementary file 3 [file JOCD-25-e71135-s003.docx]

**Supplementary table 1. Primary efficacy endpoint-The improvement rate of neck wrinkles at 4 weeks (±5 days) post-final treatment**

|  |  | **FAS** |  | **PPS** |  |
| --- | --- | --- | --- | --- | --- |
| **Indicator** | **Efficacy** | **ANN0032 (N = 78)** | **Hearty®**  **(N = 78)** | **ANN0032 (N = 78)** | **Hearty®**  **(N = 78)** |
| **4 weeks (±5 days) post-final treatment** | **Improved, n(%)** | 65(83.3) | 56(71.8) | 65 (84.4) | 56 (74.7) |
|  | **Unimproved, n (%)** | 13(16.7) | 22 (28.2) | 12 (15.6) | 19 (25.3) |
|  | **Intergroup difference in improvement rate and 95%CI** | 11.54% (-3.29%-26.37%) | | 9.75%  (-4.83%-24.33%) | |
|  | **Test statistic** | 3.17 | | 2.96 | |
|  | **P-value** | 0.002 | | 0.002 | |
| **Sensitivity analysis** | | | | | |
| FAS missing data via valid imputation | **Improved, n(%)** | 66 (84.6) | 59 (75.6) | 65 (84.4) | 56 (74.7) |
|  | **Unimproved, n (%)** | 12 (15.4) | 19 (24.4) | 12 (15.6) | 19 (25.3) |
|  | **Intergroup difference in improvement rate and 95%CI** | 8.97% (-5.26%-23.21%) | | 9.75% (-4.83%-24.33%) | |
|  | **Test statistic** | 2.91 | | 2.96 | |
|  | **P-value** | 0.002 | | 0.002 | |
| FAS missing data were not imputed | **Improved, n(%)** | 65 (84.4) | 56 (74.7) | 65 (84.4) | 56 (74.7) |
|  | **Unimproved, n (%)** | 12 (15.6) | 19 (25.3) | 12 (15.6) | 19 (25.3) |
|  | **Intergroup difference in improvement rate and 95%CI** | 9.75% (-4.83%-24.33%) | | 9.75% (-4.83%-24.33%) | |
|  | **Test statistic** | 2.96 | | 2.96 | |
|  | **P-value** | 0.002 | | 0.002 | |

Note: In the Full Analysis Set (FAS), missing data were imputed using the worst-case imputation method (assuming treatment failure). The 95% confidence interval (CI) for the difference in efficacy rates was calculated using the normal approximation method with Cochran-Mantel-Haenszel (CMH) weighting, where the CMH method was weighted according to trial center.
