# Supplementary material for: Efficacy and Safety of Sodium Hyaluronate Injection for Moderate‐To‐Severe Neck Wrinkles: A Multicenter, Single‐Blind, Randomized, Parallel‐Controlled Trial
Source: J Cosmet Dermatol. 2026 Aug 27;25(9):e71135. doi: 10.1111/jocd.71135 (PMC13522398; doi:10.1111/jocd.71135)
Supplement: Supplementary file 5 [file JOCD-25-e71135-s005.docx]

**Supplementary table 3. Secondary efficacy endpoint-Temporal dynamics of neck wrinkle improvement score**

|  |  | **FAS** |  | **PPS** |  |
| --- | --- | --- | --- | --- | --- |
| **Indicator** | **Efficacy** | **ANN0032 (N = 78)** | **Hearty®**  **(N = 78)** | **ANN0032 (N = 78)** | **Hearty®**  **(N = 78)** |
| **Week 4 (±5 days) following the initial treatment** | **Improved, n(%)** | 75(97.4) | 75 (98.7) | 75(97.4) | 74(98.7) |
|  | **Unimproved, n (%)** | 2 (2.6) | 1(1.3) | 2 (2.6) | 1 (1.3) |
|  | **Intergroup difference in improvement rate and 95%CI** | -1.28%(-5.66%-3.10%) | | -1.26% (-5.66%-3.14%) | |
|  | **Test statistic** | 0.38 | | 0.38 | |
|  | **P-value** | 1.00 | | 1.00 | |
| **Week 4 (±5 days) post-final treatment** | **Improved, n(%)** | 75(97.4) | 75(100.0) | 75(97.4) | 75(100.0) |
|  | **Unimproved, n (%)** | 2 (2.6) | 0 (0.0) | 2 (2.6) | 0 (0.0) |
|  | **Intergroup difference in improvement rate and 95%CI** | -2.60% (-6.15%-0.96%) | | -2.60% (-6.15%-0.96%) | |
|  | **Test statistic** | 0.25 | | 0.26 | |
|  | **P-value** | 0.50 | | 0.50 | |
| **Week 12 (±7 days) post-final treatment** | **Improved, n(%)** | 74(97.4) | 75(100.0) | 74(97.4) | 75(100.0) |
|  | **Unimproved, n (%)** | 2 (2.6) | 0(0.0) | 2 (2.6) | 0(0.0) |
|  | **Intergroup difference in improvement rate and 95%CI** | -2.63%(-6.23%-0.97%) | | -2.63% (-6.23%-0.97%) | |
|  | **Test statistic** | 0.25 | | 0.25 | |
|  | **P-value** | 0.50 | | 0.50 | |
| **Week 24 (±15 days) post-final treatment** | **Improved, n(%)** | 72(96.0) | 74(98.7) | 72 (96) | 74 (98.7) |
|  | **Unimproved, n (%)** | 3 (4.0) | 1(1.3) | 3 (4.0) | 1(1.3) |
|  | **Intergroup difference in improvement rate and 95%CI** | -2.67%(-7.81%-2.47%) | | -2.67%(-7.81%-2.47%) | |
|  | **Test statistic** | 0.25 | | 0.25 | |
|  | **P-value** | 0.62 | | 0.62 | |
